# *Pantoea* Bacteria Isolated from Three Thrips (*Frankliniella occidentalis*, *Frankliniella intonsa*, and *Thrips tabaci*) in Korea and Their Symbiotic Roles in Host Insect Development

**DOI:** 10.4014/jmb.2301.01018

**Published:** 2023-03-13

**Authors:** Gahyeon Jin, Yonggyun Kim

**Affiliations:** Department of Plant Medicals, Andong National University, Andong 36729, Republic of Korea

**Keywords:** Thrips, *Pantoea*, symbiosis, identification, development

## Abstract

Gut symbionts play crucial roles in host development by producing nutrients and defending against pathogens. Phloem-feeding insects in particular lack essential nutrients in their diets, and thus, gut symbionts are required for their development. Gram-negative *Pantoea* spp. are known to be symbiotic to the western flower thrips (*Frankliniella occidentalis*). However, their bacterial characteristics have not been thoroughly investigated. In this study, we isolated three different bacteria (BFoK1, BFiK1, and BTtK1) from *F. occidentalis*, *F. intonsa*, and *T. tabaci*. The bacterial isolates of all three species contained *Pantoea* spp. Their 16S rRNA sequences indicated that BFoK1 and BTtK1 were similar to *P. agglomerans*, while BFiK1 was similar to *P. dispersa*. These predictions were supported by the biochemical characteristics assessed by fatty acid composition and organic carbon utilization. In the bacterial morphological analysis, BFoK1 and BTtK1 were distinct from BFiK1. All these bacteria were relatively resistant to tetracycline compared to ampicillin and kanamycin, in which BFoK1 and BTtK1 were different from BFiK1. Feeding ampicillin (100,000 ppm) reduced the bacterial density in thrips and retarded the development of *F. occidentalis*. The addition of BFoK1 bacteria, however, rescued the retarded development. These findings indicate that *Pantoea* bacteria are symbionts to different species of thrips.

## Introduction

Despite the abundant intestinal and intracellular bacteria in insects, the functional associations between them and their symbiotic bacteria are understood in only a few insect species [1]. Some insects, such as aphids harboring *Buchnera* species, possess obligate endosymbionts, to which the host insect provides specific nutrients [2]. These obligate symbionts are known to be transferred frequently by vertical transmission, as exemplified in aphids [3]. Other insects possess facultative symbionts that are beneficial but not essential to their host, as seen in *Hamiltonella defensa* aphids against parasitoid wasps [4]. Such facultative bacteria are usually a dominant species in the gut, as in the case of hemipteran triatomines [5], and are simply transmitted to the next generation by coprophagy [6].

The western flower thrips (WFT), *Frankliniella occidentalis*, is a globally invasive agricultural pest of significant importance [7]. It causes direct damage to crops by feeding and/or ovipositing on leaves and fruits, which eventually results in a decreased yield and low market value [8]. These insects also transmit plant pathogenic tospoviruses (including tomato spotted wilt virus), which cause significant economic damage to plants [9]. In addition, WFTs exhibit a specific association with two bacteria, BFo1 and BFo2 [10-12]. Interestingly, BFo1 and BFo2 have been isolated from different geographic locations, including California, Hawaii, Germany, the Netherlands, and the United Kingdom [10-12]. This suggests a symbiotic rather than a transient relationship of the bacteria with the thrips. BFo1 is closely related to *Erwinia* spp., whereas BFo2 is a novel species related to *Pantoea* [12]. Vertical transmission from parent to progeny appears to be through feeding, probably via plant material contaminated with the frass of adults [13]. However, it is unclear whether the symbiotic relationship between the bacteria and thrips is mutualistic or commensal.

Since the first observation of *F. occidentalis* in 1993, it has spread to most of the cultivated areas in Korea [14]. The species WFT is further diversified into two strains (WFTL and WFTG) [15], with most Korean populations belonging to WFTG, while North American populations consist of both strains [16]. This genetic variation in host insects suggests a variation in thrips-associated bacteria. In addition, the bacterial species in other thrips, including *F. intonsa* and *Thrips tabaci*, which co-localize with *F. occidentalis* in the same crops [17], need to be clarified.

In this study, we isolated and identified the bacteria from three species of thrips (*F. occidentalis*, *F. intonsa*, and *T. tabaci*) based on their morphological, physiological, and molecular characteristics. The symbiotic relationship of the bacterial isolates with *F. occidentalis* was then assessed by analyzing host development after antibiotic treatment compared to controls.

## Materials and Methods

### Collection and Rearing of Thrips

Adult *F. occidentalis* thrips were donated by Bio Utility, Inc. (Korea). Adult onion thrips (*T. tabaci*) and flower thrips (*F. intonsa*) were collected from a Welsh onion (*Allium fistulsum* L.) field in Suanbo, Korea. All thrips species were reared in a laboratory under constant temperature conditions of 25 ± 1°C, 16:8 h (L:D) photoperiod, and 60 ± 5% relative humidity. *F. occidentalis* and *F. intonsa* were reared with sprouted bean seed kernels (*Phaseolus vulgaris* L.), whereas *T. tabaci* was reared on fresh onion leaves. Under the laboratory conditions, *F. occidentalis* underwent two larval instar stages (L1 and L2).

### Isolation of Symbiotic Bacteria

Bacterial isolation from the thrips followed the method described earlier by de Vries *et al*. [10]. Briefly, a sample consisted of five adults and five larvae in each thrips species, and was placed in a 1.5 ml tube. The thrips were surface-sterilized by sequentially soaking them in 70% ethanol for 1 min and 5% sodium chlorite for 1 min. After rinsing three times with sterilized water, each thrips sample was then homogenized in 100 μl of Tris/EDTA buffer (10 mM Tris, 1 mM EDTA, pH 7.6). The thrips homogenate was spread on Luria-Bertani (LB) agar medium and incubated for 24 h at 25°C.

### Molecular Identification of Bacterial Isolates

Total genomic DNA was extracted from the isolated bacteria using a QIAprep Spin Miniprep Kit (Qiagen, USA) for genomic identification. The 16S rRNA region was amplified by polymerase chain reaction (PCR) using the forward (5-GAAGAGTTTGATCATGGCTC-3) and reverse (5-AAGGAGGTGATCCAGCCGCA-3) primers reported by Tailliez *et al*. [18]. The extracted genomic DNA was used as a template for PCR amplification with the 16S rRNA primer pair with 35 cycles under the following conditions: 1 min at 94°C for denaturation, 30 s at 52°C for annealing, and 1 min at 72°C for extension. The resulting PCR product was bi-directionally sequenced by Macrogen (Korea). The resulting nucleotide sequences were uploaded to GenBank (https://www.ncbi.nlm.nih.gov) to determine the matched bacterial species using the BLASTn search engine.

### Transmission Electron Microscopy Analysis of Bacterial Isolates

The bacteria isolated from the three thrips species were cultured in LB at 25°C. After overnight culture, the bacteria were attached to a 200-mesh copper grid coated with carbon-stabilizer Formvar. After negative staining with 2% phosphotungstic acid, each sample was observed by transmission electron microscopy (TEM, H-7650, Hitachi, Japan) at 12,000 × magnification to observe multiple bacteria and 80,000 × magnification to observe single bacterial cells.

### Analysis of Biochemical Characteristics of Bacterial Isolates

For the biochemical characterization of a symbiotic bacterium, Gram-stain and catalase/oxidase activity tests were performed according to the methods described by Schaad *et al*. [19]. These characteristics were used to determine the bacterial genus by comparing them to those of bacteria described in Bergey's Manual [20]. The acid production characteristics of the isolate from different carbon sources were assessed using a colorimetric method and GN microplate (Biolog, USA), and the results were compared to those of other *Pantoea* species. Fatty acid composition of the bacterial cell wall was analyzed by gas chromatography (Agilent 6890, USA).

### Antibiotic Testing

Ampicillin, kanamycin, and tetracycline were purchased from Sigma-Aldrich (Korea), and test solutions were prepared in distilled water. A paper disk (1 mm diameter) was soaked in each concentration of antibiotic solution. After culturing the test bacteria at 10^8^ CFU/ml, 100 μl of bacterial suspension was spread on LB medium. Then, a treated disc was placed on the center of the plate. After 18 h at 25°C, the inhibition zone was measured. Each treatment was replicated three times.

### Test of a Symbiotic Relationship Between a Bacterial Isolate (BFoK1) and *F. occidentalis*

Beans treated with antibiotic or bacteria were fed to the thrips. Ampicillin was used at 100,000 ppm. BFoK1 or *Escherichia coli* Top10 was cultured at 10^8^ CFU/ml. Diet beans were soaked in antibiotic solution or bacterial suspension for 1 min and used to feed the larvae. Each treated diet bean was fed to 10 larvae for 2 days. After 14 days at 25 ± 1°C, 16:8 h (L:D) photoperiod, and 60 ± 5% relative humidity, the developed adults were counted. Each treatment used 10 larvae and was replicated three times.

### Statistical Analysis

Percentage data were arcsine-transformed for normalization. The transformed data were subjected to analysis of variance (ANOVA) using PROC GLM in the SAS program [21]. The mean difference was discriminated at Type I error of 0.05 by the Fishers Least Significant Difference (LSD) test. Frequency data were analyzed by the Chi-squared test using PROC FREQ.

## Results

### Molecular Identification of Bacteria Isolated from Thrips

**Fig. 1:**
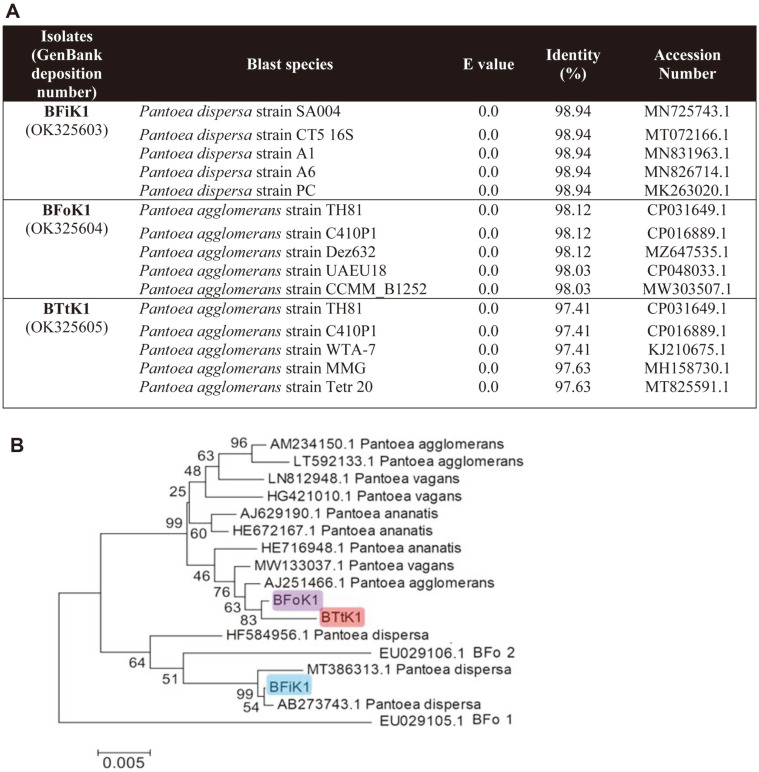
Molecular identification of thrips-associated bacterial isolates: BFoK1 from *F. occidentalis*, BFiK1 from *F. intonsa*, and BTtK1 from *Thrips tabaci*. (**A**) BLASTN search of the three isolates in GenBank using the 16S rRNA sequences. (**B**) Phylogenic analysis of the 16S rRNA sequences of the three isolates with other bacterial species in *Pantoea*. Figures on branches indicate bootstrap values with 1,000 repetitions using MEGA6.0. The scale bar indicates genetic divergence in the nucleic acid sequence.

Thrips extracts were diluted 1,000-fold and grown on LB. From the resulting colonies (about 100 colonies per plate), 10 yellow bacterial colonies were randomly isolated. The 16S rRNA sequences of the bacterial isolates were then assessed, and those (3~4 colonies per each thrips species) with a close relationship with the *Pantoea* genus were selected through a BLAST search in GenBank (Fig. 1A). None of the isolates showed similarity to the *Erwinia* genus. Biochemical identification supported the genus identification (Table S1). All three bacteria were gramnegative and motile. They exhibited negative responses in catalase and oxidase tests. The bacterial isolates from *F. occidentalis*, *F. intonsa*, and *T. tabaci* were named BFoK1, BFiK1, and BTtK1, respectively. BFoK1 and BTtK1 were the most homologous to *P. agglomerans* with 98.12 and 97.41% of 16S rRNA gene identity, respectively (Fig. 1B). BFiK1 was somewhat different and highly matched to *P. dispersa*, with 98.94% identity. When the three bacterial isolates were compared to the known thrips bacteria BFo1 and BFo2, they were clustered with BFo2 but separated from BFo1. BFoK1, BFiK1, and BTtK1 were deposited into the Korean Agricultural Culture Collection (KACC) under the accession numbers KACC 92380P, KACC 92379P, and KACC 92381P.

### Morphological and Physiological Characteristics of Bacterial Isolates

**Fig. 2:**
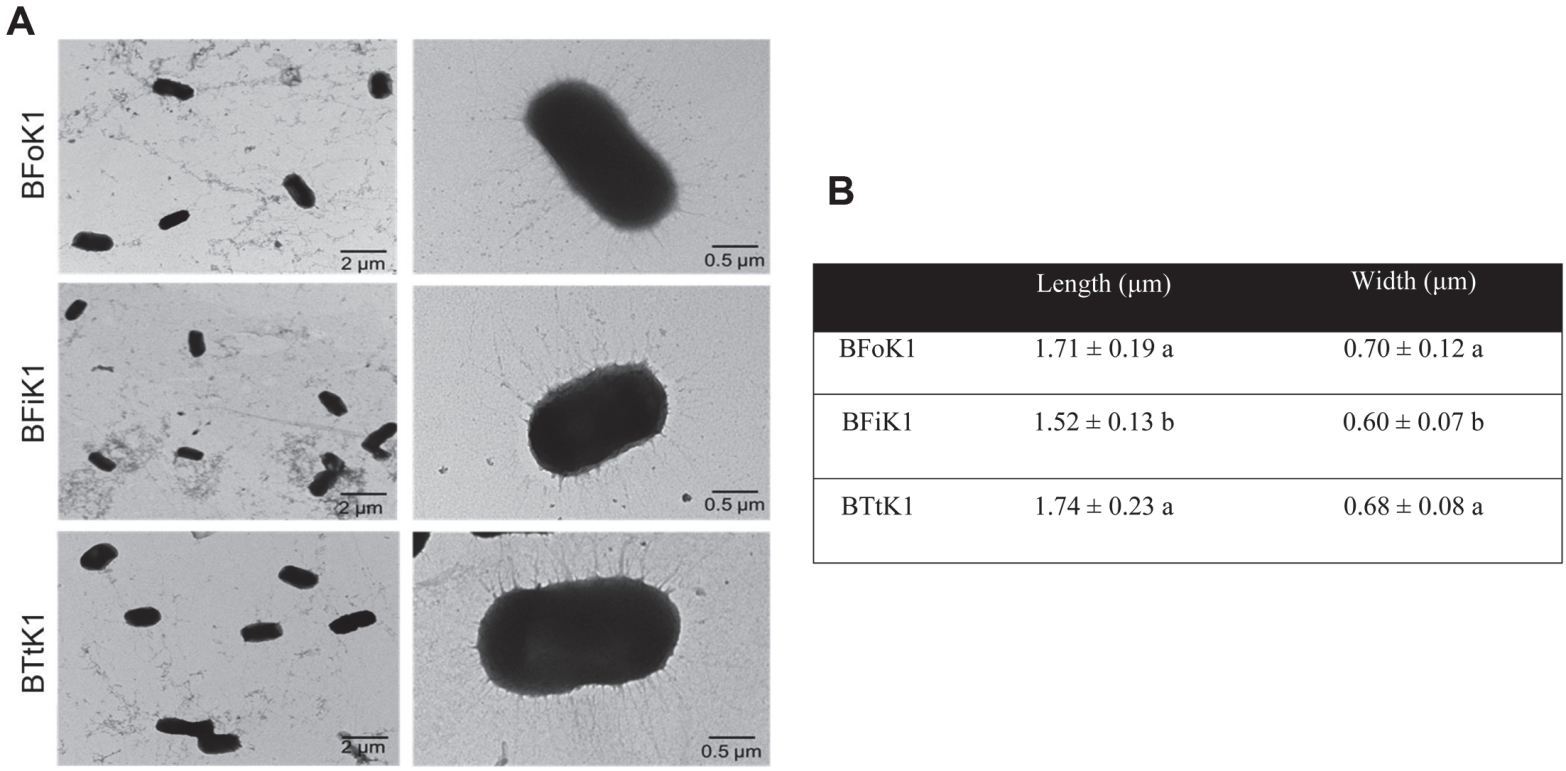
Microscopic analysis of three thrips-associated bacterial isolates: BFoK1 from *F. occidentalis*, BFiK1 from *F. intonsa*, and BTtK1 from *Thrips tabaci*. (**A**) TEM photos indicating group or individual bacterial structures. (**B**) Comparison of bacterial size. Each measurement used 10 different bacteria. Different letters followed by the figures indicate significant differences between the means at Type I error of 0.05 (LSD test).

The three bacterial isolates were observed by TEM (Fig. 2). All were rod-shaped and had numerous pili (Fig. 2A). BFoK1 and BTtK1 were similar in size, but BFiK1 was smaller (Fig. 2B).

Carbon utilization was assessed using a 96-well plate containing different substrates provided by Biolog Microbial Identification (Table 1). All three bacterial isolates exhibited high similarity (> 84.7%) to *Pantoea* spp. in carbon utilization, in which BFoK1 and BTtK1 were highly similar (86.7 and 86.1%) to *P. agglomerans*, and BFiK1 was highly similar (93.0%) to *P. dispersa*.

**Fig. 3:**
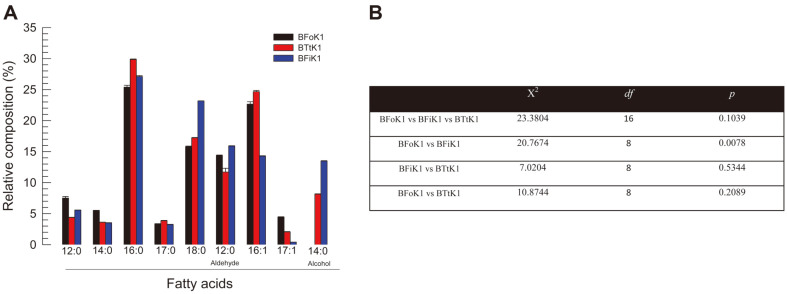
Fatty acid composition of three thrips-associated bacterial isolates: BFoK1 from *F. occidentalis*, BFiK1 from *F. intonsa*, and BTtK1 from *Thrips tabaci*. (**A**) Relative composition of seven fatty acids: lauric acid (12:0), dodecanal (12:0 aldehyde), myristic acid (14:0), myristyl alcohol (14:0 alcohol), palmitic acid (16:0), hexadecenoic acid (16:1), margaric acid (17:0), heptadecenoic acid (17:1), and stearic acid (18:0). (**B**) Chi-squared test of fatty acid composition in bacterial isolates.

The fatty acid composition of the bacterial cell membranes of the three bacteria was assessed (Fig. 3). A total of nine fatty acids were compared, of which seven were saturated and two were unsaturated (Fig. 3A). The three major fatty acids present at > 20% in the bacteria were palmitic acid (16:0), stearic acid (18:0), and palmitoleic acid (16:1). This overall pattern was observed in all three bacteria. However, there was a significant variation in fatty acid composition between BFoK1 and BFiK1.

**Fig. 4:**
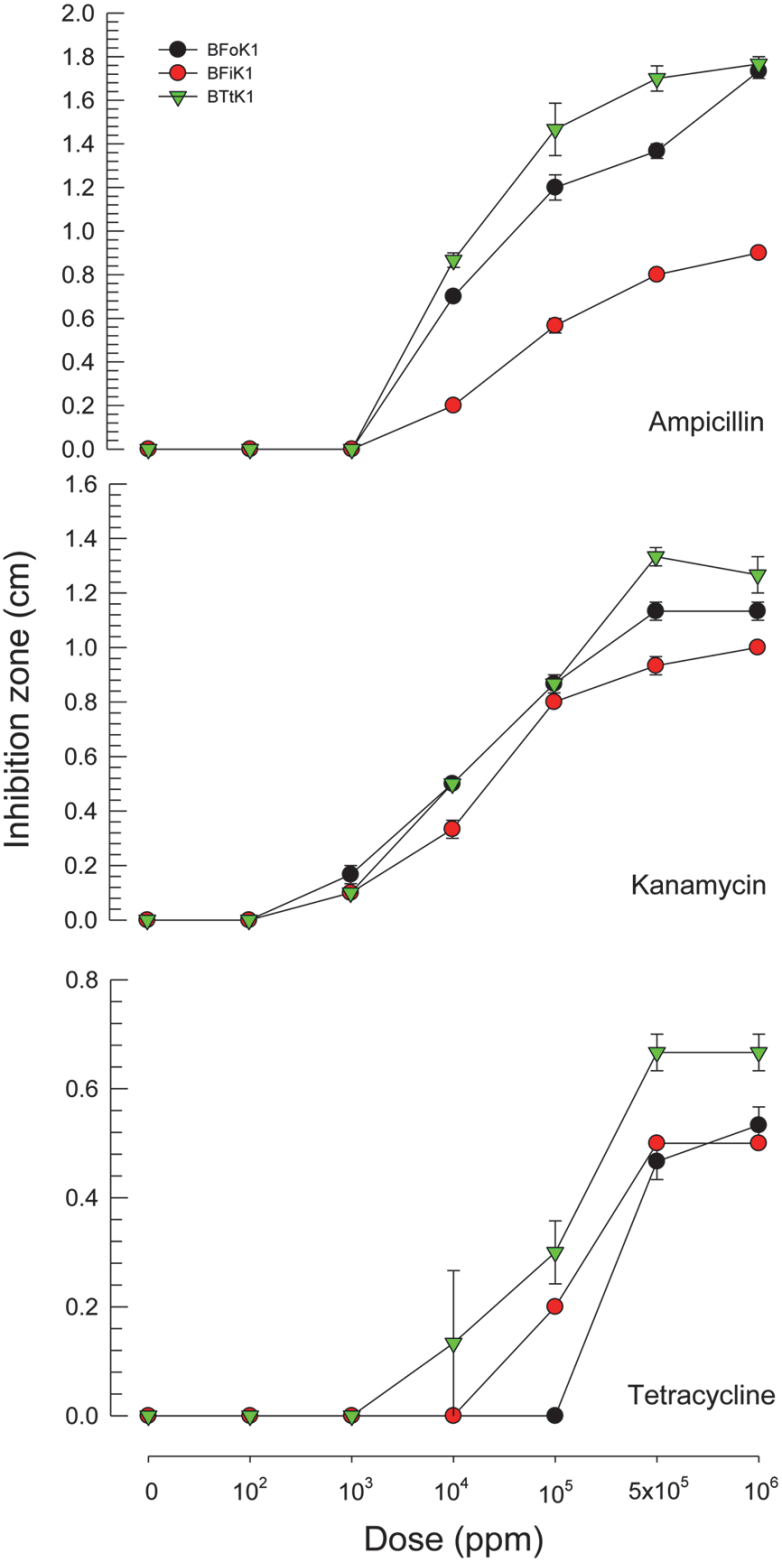
Susceptibility of the three bacteria derived from three thrips to different concentrations of antibiotics. The bacteria were derived from *F. occidentalis* (BFoK1), *F. intonsa* (BFiK1), and *Thrips tabaci* (BTtK1). Each treatment was replicated three times.

The susceptibility to antibiotics of the three bacteria was compared (Fig. 4). At high concentrations above 5×10^5^ ppm, all three bacteria were highly susceptible. However, the minimal inhibition concentration (MIC) for the three antibiotics was different (Table S2). All three bacteria were relatively tolerant to tetracycline compared to ampicillin or kanamycin. Among the three bacteria, susceptibility to each antibiotic varied (Table 2).

### Physiological Significance of BFoK1 in *F. occidentalis* Development

**Fig. 5:**
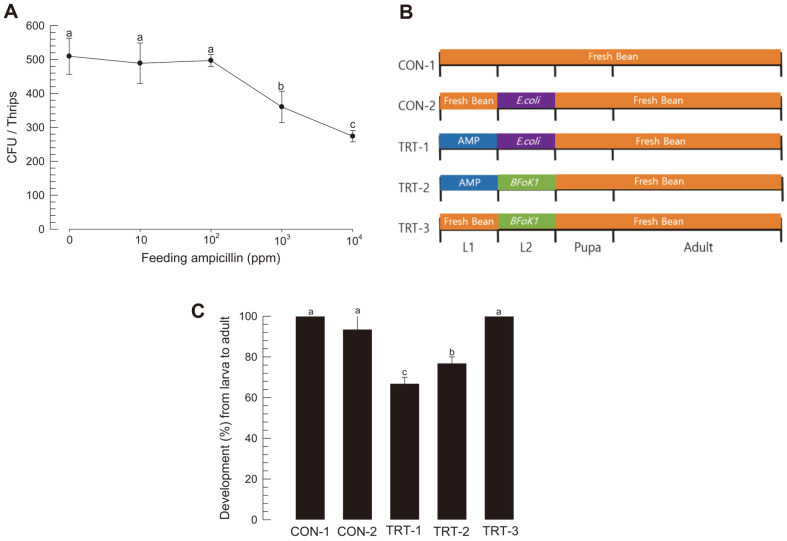
Influence of BFoK1 on the development of *F. occidentalis*. (**A**) Different feeding regimes of ampicillin and BFoK1 to larvae. *E. coli* was used as the control for bacterial feeding. (**B**) Toxicity of ampicillin on gut bacteria at different doses. Each treatment used five thrips and was replicated three times. (**C**) Rescue of ampicillin-treated thrips (‘TRT-1’) by the addition of BFoK1 (‘TRT-2’). Each treatment used 10 larvae and was replicated three times. Different letters above the standard deviation bars indicate significant differences among the means at Type I error of 0.05 (LSD test).

To test the hypothesis regarding the requirement of BFoK1 in the development of *F. occidentalis*, ampicillin (AMP, TRT-1) was fed to young larvae (Fig. 5). Controls (CON-1) were set by treating larvae without any bacteria (Fig. 5A). To recover from antibiotic treatment, BFoK1 was added to the diet (TRT-2), in which the control (CON-2) was *E. coli*. BFoK1 was added to the control larvae (TRT-3). As expected, AMP feeding reduced bacterial density in the thrips (Fig. 5B). Even though the addition of *E. coli* or BFoK1 did not adversely affect thrips development, AMP treatment significantly interfered with thrips development from larvae to adult (Fig. 5C). Moreover, the addition of BFoK1 to the antibiotic treatment significantly rescued the slowed development.

## Discussion

The symbiotic gut bacteria of *F. occidentalis* and related thrips were previously isolated in different countries. Here, we isolated three different bacteria, BFoK1, BFiK1, and BTtK1, in Korea. These isolates were classified into the *Pantoea* genus and their 16S rRNA sequences were phylogenetically associated with BFo2, but not with BFo1 isolated from *F. occidentalis*. In *F. occidentalis*, BFo1 was closely related to *Erwinia* spp., whereas BFo2 was related to *Pantoea* [12]. Biochemical tests for carbon utilization supported the genus identification of the three Korean isolates as *Pantoea*. However, the 16S rRNA sequences indicated that BFoK1 and BTtK1 were likely to be *P. agglomerans*, and BFiK1 was *P. dispersa*. The morphological characteristics from the TEM analysis and carbon utilization tests indicated a difference between BFiK1 and BFoK1 and BTtK1. Fatty acid composition analysis also showed differences between BFoK1 and BFiK1.

This is the first report on the isolation of *P. dispersa* from *F. intonsa*. Even though *F. intonsa* shares similar hosts and habitats with *F. occidentalis*, no information is available on the symbiotic gut bacteria of *F. intonsa*. The bacterial genus *Pantoea* comprises yellow-colored gram-negative bacteria and occurs in various habitats, including plants, animals, soil, and water [22]. In plants, *Pantoea* is primarily recognized as a pathogenic genus. P. ananatis infects various crops, including onions, by causing serious rotting symptoms in the middle of onion bulb in Korea [23]. This bacterium is carried by another thrip, *F. fusca* [24]. *P. dispersa* was also reported as a pathogen in rice and onion production in East Asia [25, 26]. This suggests that *F. intonsa* may transmit the bacterial pathogen to crops in addition to tomato spotted wilt virus (TSWV) [16].

BFoK1 and BTtK1 were similar to *P. agglomerans*. The first symbiont gut bacterium, from genus *Erwinia* (*Enterobacteriaceae*), was isolated from *F. occidentalis* [27]. Possibly *P. agglomerans*, it is consistently present in *F. occidentalis* [10]. It is localized in the thrips gut at 10^5^ bacteria at the L2 stage and transmitted to progeny via the leaves that both the adults and larvae eat [13]. The presence of bacteria was reported to facilitate larval development and increase fecundity [28]. A similar type of bacteria might be localized in *T. tabaci* due to the similar life cycle and polyphagous feeding behavior, although *T. tabaci*, unlike *F. occidentalis*, is thelytokous (unfertilized diploid eggs becoming females) in the Korean population [29]. Like *F. occidentalis*, *T. tabaci* contained a dominant bacterial species identified as an *Erwinia* species, but its biochemical characteristics and 16S rDNA sequence differed from that of the bacteria present in *F. occidentalis* [30]. Later, *T. tabaci* was found to transmit two *Pantoea* bacteria (P. ananatis and *P. agglomerans*) to onions to cause rotting disease [31]. This suggests a functional association between the bacteria and *T. tabaci*. Our current study to identify *P. agglomerans* from *T. tabaci* further supported a bacterial association with thrips. Our Korean population of *F. occidentalis* did not include BFo1. In contrast, a thrips population in the UK did not harbor BFo2 but did contain BFo1. A field survey in California [32] suggested that the occurrence of symbiotic bacteria in *F. occidentalis* was influenced by environmental conditions, such as altitude, temperature, and precipitation. In this study, dual bacterial infections with BFo1 and BFo2 were found in 50% of the population while single infections were found in 32%, and no infections were found in 18%. The occurrence of BFo1 increased with decreases in temperature and increases in precipitation. Furthermore, bacterial competition was found between BFo1 and BFo2 in the same habitat in the thrips gut regarding the preference of thrips host types [4]. Thus, a single (BFo2) infection of Korean populations of *F. occidentalis* and *T. tabaci* may be explained in the local environments in Korea specifically favored by BFo2 type, as well as a specific genetic variation of the thrips in Korea.

BFoK1 facilitated the immature development of *F. occidentalis*. The gut bacteria were reported in different local populations from Hawaii, California, Germany, and the Netherlands, and these local populations had a unique phylogenic integrity, suggesting their symbiotic relationship [11]. The symbiotic bacteria were localized in the specific hindgut and their association was maintained in successive generations for more than two years, indicating a permanent association between the bacteria and the thrips [10]. However, the bacterium is a facultative symbiont since it is able to grow outside of the thrips. There is no evidence of the vertical transmission of the bacteria from mother to progeny via eggs in *F. occidentalis* [28]. Thus, the young larvae uptake the bacteria from the habitat shared with their parents and within the thrips, and the introduced bacteria grow during feeding stages [13]. Our current study indicates that the bacteria facilitate the growth of the host thrips, *F. occidentalis*. However, the nutritional benefit of symbiotic bacteria in facilitating host growth remains unsolved in this study and needs to be clarified in the future. Another benefit from symbiotic bacteria may be bacterial secondary metabolites. *P. agglomerans* identified from BFoK1 is also found in the gut of locusts and mosquitoes. The locusts have adapted to using guaiacol produced by the bacteria to initiate synchronized swarming [33]. A genetically modified strain of the symbiotic bacteria producing antimalarial effector molecules reduced the prevalence of the malaria-causing organism (*Plasmodium*) by up to 98% [34]. Although this study did not test the symbiotic roles of BTtK1 or BFiK1, they might also influence the development of their hosts. This speculation needs to be assessed in future study.

## Supplemental Materials

Supplementary data for this paper are available on-line only at http://jmb.or.kr.

## Figures and Tables

**Table 1:** Characteristics of the carbon source utilization of symbiotic bacteria isolated from different thrips and other *Pantoea* spp.

Group	Carbon source	Response^1^ of *Pantoea* spp.^2^							

		BTtK1	BFoK1	BFiK1	Pd	Pa	Pe	Paa	Pc
I	D-Sorbitol, D-Lactic Acid Methyl Ester, D-Malic Acid, α- Hydroxy-Butyric Acid, α-Keto-Butyric Acid, Acetoacetic Acid, Propionic Acid, Acetic Acid, Formic Acid	-	±	±	±	±	±	±	±
II	D-Raffinose, α-D-Lactose, L-Fucose, D-Aspartic Acid, Gelatin	-	-	-	-	-	-	±	-
III	D-Serine, L-Pyroglutamic Acid, p-Hydroxy-Phenylacetic Acid	-	-	±	-	-	-	-	-
IV	L-Lactic Acid	-	+	+	+	+	+	+	+
V	Dextrin, D-Maltose, D-Trehalose, D-Cellobiose, Gentiobiose, Sucrose, D-Turanose, D-Melibiose, β- Methyl-D-Glucoside, D-Salicin, α-D-Glucose, DMannose, D-Fructose, 3-Methyl Glucose, D-Mannitol, LArginine, L-Histidine, L-Serine, Glucuronamide, Quinic Acid, Methyl Pyruvate, Tween 4-0, γ-Amino- Butyric Acid	±	±	±	±	±	±	±	±
VI	N-Acetyl-D-Mannosamine, N-Acetyl-D-Galactosamine, N-Acetyl Neuraminic Acid, , L-Rhamnose, D-Glucose- 6- PO4, L-Alanine, Citric Acid, Bromo-Succinic Acid,	+	±	±	±	±	±	±	±
VII	N-Acetyl-D-Glucosamine, D-Galactose, Inosine, DArabitol, Myo-inositol, Glycerol, D-Fructose- 6-PO4, Glycyl-L-Proline, L-Aspartic Acid, L-Glutamic Acid, DGalacturonic Acid, D-Gluconic Acid, D-Glucuronic Acid, Mucic Acid, D-Saccharic Acid, L-Malic Acid	+	±	±	+	+	+	+	+
VIII	Stachyose, α-Keto-Glutaric Acid	+	-	-	-	-	-	-	-
	Similarity (%) to BTtK1	100			84.7	86.1	84.8	81.9	84.7
	Similarity (%) to BFoK1		100		84.7	84.7	84.7	79.1	84.7
	Similarity (%) to BFiK1			100	93.0	93.0	90.2	90.2	93.0

^1^Positive (+) and negative (-) responses. ‘±’ indicates borderline response between + and -.

^2^Pd, Pa, Pe, Paa, and Pc represents *Pantoea dispersa*, *Pantoea agglomerans*, *Pantoea eucrina*, *Pantoea ananatis* pv. *ananatis*, and *Pantoea cypripedii*.

**Table 2:** ANOVA of the susceptibility of the three bacteria (BAC) derived from three thrips to different concentrations (CONC) of antibiotics.

Source	*df*	SS	MS	*F*	*P*

Ampicillin					

BAC	2	2.38	1.18	267.43	<.0001
Fo vs Tt	(1)	(2.19)	2.19	493.71	<.0001
Fo+Tt vs Fi	(1)	(0.18)	0.18	41.14	<.0001
CONC	6	22.25	3.70	834.49	<.0001
BAC × CONC	12	2.35	0.195	44.10	<.0001

Kanamycin					

BAC	2	0.18	0.09	48.08	<.0001
Fo vs Tt	(1)	(0.17)	0.17	91.12	<.0001
Fo+Tt vs Fi	(1)	(0.009)	0.009	5.04	0.0301
CONC	6	14.05	2.34	1230.19	<.0001
BAC × CONC	12	0.24	0.02	10.36	<.0001

Tetracycline					

BAC	2	0.13	0.07	18.57	<.0001
Fo vs Tt	(1)	(0.07)	0.07	18.85	<.0001
Fo+Tt vs Fi	(1)	(0.07)	0.07	18.28	0.0001
CONC	6	3.58	0.60	163.33	<.0001
BAC × CONC	12	0.15	0.01	3.55	0.0011

Bacteria were derived from *F. occidentalis* (‘Fo’), *F. intonsa* (‘Fi’), and *Thrips tabaci* (‘Tt’).
