# Supplementary material for: Pantoea Bacteria Isolated from Three Thrips (Frankliniella occidentalis, Frankliniella intonsa, and Thrips tabaci) in Korea and Their Symbiotic Roles in Host Insect Development
Source: J Microbiol Biotechnol. 2023 Mar 13;33(6):745–52. doi: 10.4014/jmb.2301.01018 (PMC10331939; doi:10.4014/jmb.2301.01018)
Supplement: Supplementary file 1 [file jmb-33-6-745-supple.pdf]

J. Microbiol. Biotechnol.
https://doi.org/10.4014/jmb.2301.01018

Supplementary Tables

Table S1. Characteristics used to identify the genus of the symbiotic bacteria isolated from different thrip species.

	BFiK1	BFoK1	BTtK1	Genus *Pantoea*
Gram test	-	-	-	-
Mobility	+	+	+	+
Catalase	-	-	-	-
Oxidase	-	-	-	-

Table S2. Minimal inhibitory concentration (MIC) of three different antibiotics against three bacterial isolates from thrips.

Antibiotics	MIC (ppm)		
	BFiK1	BFoK1	BTtK1
Ampicillin	10000	1000	10000
Kanamycin	1000	1000	1000
Tetracycline	500000	100000	100000
